# Discriminating Multi-Species Populations in Biofilms with Peptide Nucleic Acid Fluorescence *In Situ* Hybridization (PNA FISH)

**DOI:** 10.1371/journal.pone.0014786

**Published:** 2011-03-29

**Authors:** Carina Almeida, Nuno F. Azevedo, Sílvio Santos, Charles W. Keevil, Maria J. Vieira

**Affiliations:** 1 Institute for Biotechnology and Bioengineering (IBB), Centre of Biological Engineering, Universidade do Minho, Campus de Gualta, Braga, Portugal; 2 Environmental Healthcare Unit, School of Biological Sciences, University of Southampton, Southampton, United Kingdom; 3 Department of Chemical Engineering, Faculty of Engineering, University of Porto, Porto, Portugal; Instituto de Tecnologia Química e Biológica, Portugal

## Abstract

**Background:**

Our current understanding of biofilms indicates that these structures are typically composed of many different microbial species. However, the lack of reliable techniques for the discrimination of each population has meant that studies focusing on multi-species biofilms are scarce and typically generate qualitative rather than quantitative data.

**Methodology/Principal Findings:**

We employ peptide nucleic acid fluorescence in situ hybridization (PNA FISH) methods to quantify and visualize mixed biofilm populations. As a case study, we present the characterization of Salmonella enterica/Listeria monocytogenes/Escherichia coli single, dual and tri-species biofilms in seven different support materials. Ex-situ, we were able to monitor quantitatively the populations of ∼56 mixed species biofilms up to 48 h, regardless of the support material. In situ, a correct quantification remained more elusive, but a qualitative understanding of biofilm structure and composition is clearly possible by confocal laser scanning microscopy (CLSM) at least up to 192 h. Combining the data obtained from PNA FISH/CLSM with data from other established techniques and from calculated microbial parameters, we were able to develop a model for this tri-species biofilm. The higher growth rate and exopolymer production ability of *E. coli* probably led this microorganism to outcompete the other two [average cell numbers (cells/cm^2^) for 48 h biofilm: *E. coli* 2,1×10^8^ (±2,4×10^7^); *L. monocytogenes* 6,8×10^7^ (±9,4×10^6^); and *S. enterica* 1,4×10^6^ (±4,1×10^5^)]. This overgrowth was confirmed by CSLM, with two well-defined layers being easily identified: the top one with *E. coli*, and the bottom one with mixed regions of *L. monocytogenes* and *S. enterica*.

**Significance:**

While PNA FISH has been described previously for the qualitative study of biofilm populations, the present investigation demonstrates that it can also be used for the accurate quantification and spatial distribution of species in polymicrobial communities. Thus, it facilitates the understanding of interspecies interactions and how these are affected by changes in the surrounding environment.

## Introduction

According to Costerton et al. [1], a biofilm is “a functional consortium of microorganisms attached to a surface and is embedded in the extracellular polymeric substances (EPS) produced by the microorganisms”. Biofilm formation has been recognized as a well-know strategy used by microorganisms to survive within hostile environments, such as those encountered in host tissues (antibodies, phagocytes, etc.) or on inert surfaces exposed to inhospitable conditions (UV light, desiccation, heat, cold, shear forces). Likewise, organisms within a biofilm are far more resistant to antimicrobial agents than those in suspension [2].

It is known that, in nature, most biofilms are composed of multiple species [3]. These multispecies biofilms are responsible for significant problems in many areas, such as the corrosion of liquid-carrying vessels, biofouling in drinking water distribution systems, contamination of food processing environments, persistent and recurrent infections and device-related infections in humans [4], [5], [6], [7], [8], [9], [10], [11]. On the other hand, they have been successfully employed to treat wastewater and coculture in biofilms is now being used to improve power generation in microbial fuel cells [12], [13]. To fully characterize and understand these systems, it is necessary to spatially discriminate between one or more populations, and as such, widely used methods in the biofilm area, such as cristal violet (CV), SYTO9/propidium iodide fluorochrome uptake and 4′,6-diamidino-2-phenylindole (DAPI) staining, are insufficient due to their non-specific nature. Consequently, to overcome this problem several researchers have been using different approaches, such as mutants expressing green fluorescent protein (GFP) [14], [15], [16], fluorescently labeled antibodies [17], [18], [19] and fluorescence *in situ* hybridization (FISH) [20], [21], [22]. Nevertheless, these methods present severe limitations. For instance, for fluorescently labeled antibodies, cross reactivity and the need to cultivate the bacterial strains to raise antisera, imply that the method is labour-intensive and suffers from lack of specificity [21]. GFP has been successfully applied to the mixed-biofilm studies, but the use of this reporter molecule is restricted by several environmental factors, such as oxygen requirement for GFP chromophore formation, and its poor fluorescence at low pH [14]. It is also necessary to previously develop the strain expressing the protein, which does not allow for natural samples analysis and is labour-intensive. FISH has emerged as a molecular alternative because it can be applied to environmental samples and is based in phylogenetic markers at 16 or 23S rRNA, that are less influenced by the growth-condition [21], [23], [24]. Traditionally, FISH uses labeled DNA probes for the *in situ* identification of microorganisms by hybridization to ribosomal RNA. DNA probes however implied that the method suffers from limitations related to cell permeability, hybridization affinity and target site accessibility, leading to poor signal-to-noise ratios and lack of target site specificity and sensitivity [21], [23], [25].

For certain applications, particularly in clinical diagnostics, food safety and microbial ecology, some authors have showed that FISH limitations could be overcome by the use of peptide nucleic acid (PNA) probes [26], [27], [28], [29]. PNA is a synthetic DNA analogue that presents a quicker and stronger binding to DNA/RNA attributed to the lack of charge repulsion between the neutral PNA strand and the complementary RNA strand [30]. Consequently the probes used for PNA FISH are shorter, usually consisting of 15 bases, and present higher specificity and sensitivity than conventional DNA probes. Hybridization can be performed efficiently under low salt concentrations, which destabilize the rRNA secondary structure, resulting in an improved access to the target sequences. The hydrophobic nature of the PNA molecule allows an easy penetration in the cell, and theoretically a better diffusion through the biofilm matrix [27]. The combination of this method with confocal laser scanning microscopy (CLSM) allows the study of spatial organization and changes of specific members of complex microbial populations without disturbing the biofilm structure [31], [32]. Despite the potential of PNA probes, there are just a few studies regarding their application to biofilms (for selected examples see [28], [31], [33]), and even these are limited to the detection/identification of populations within a biofilm and to assess the spatial organization.

In here, we evaluate and validate PNA FISH to quantify and characterize the initial adhesion and biofilm formation of three microorganisms, *Salmonella enterica*, *Escherichia coli* and *Listeria monocytogenes*. The method is compared against CV and culture techniques for seven different support materials, either in mono-, dual- or tri-species biofilms. A global multispecies biofilm development model for a biofilm formed from these three microorganisms based on the collected information is then proposed.

## Materials and Methods

### Culture maintenance

All chemicals were obtained from Sigma, unless otherwise stated. *S. enterica* ATCC 13076, *E. coli* ATCC 25922 and *L. monocytogenes* ATCC 15313 were maintained on Tryptic Soy Agar (TSA) (VWR, Portugal) at 37°C and streaked onto fresh plates every 24 hours.

### Biofilm formation

For all experiments, cells were grown overnight (16 to 18 h) in Tryptic Soy Broth (TSB) (VWR, Portugal), at 37°C, 120 rpm. Cell concentration was then assessed by optical density (O.D.) and this initial culture was further diluted in order to obtain a final concentration of approx. 10^8^ total cells/ml. After homogenization, 6 ml of the suspension were dispensed into each well of a six-well tissue culture plate (Orange Scientific, Braine-l'Alleud, Belgium) containing coupons of different materials(glass, polypropylene [PP], polyethylene [PE], polyvinyl chloride [PVC], copper, silicone rubber [Sil] and stainless steel [Steel]), prepared as previously described [34]. The tissue culture plates were then placed in an incubator (Shell Lab, Oregon) at 21°C, in standing culture. At different sampling times (2, 4, 6, 24 and 48 h), coupons were removed from the tissue plates, washed three times in 10 ml of sterile distilled water and biofilm formation was assessed by plate counts, CV assay, PNA FISH or DAPI staining as described below. This experiment was performed in triplicate for each one of the species used, and for all the possible combinations between species (*E. col*i/*S. enterica*; *E. coli*/*L. monocytogenes*; *S. enterica*/*L. monocytogenes* and *E. coli*/*S. enterica*/*L. monocytogenes*).

We also determined the growth rates for each strain at 21°C on TSB. For this, cells were grown overnight as described above, diluted 1 to 100, incubated at 21°C, 120 rpm, and the O. D. was measured along the time untill the stationary stage.

### Cultivability assessment

After washing, the coupons with biofilm were placed in a new six-well tissue culture plate with 6 ml of sterile distilled water and sonicated with a 5-s burst at 25% amplitude (GEX 400 ultrasonic processor; Sigma).

Next, 100 µl samples were taken to assess cultivability, by plating the appropriate dilutions in agar plates, in triplicate. For these cultivability assays, two different media were used: MacConkey agar (Liofilchem, Italy), that discriminates between *S. enterica* and *E. coli* based on each species ability to consume lactose, and the Oxford agar (Liofilchem, Italy) for *L. monocytogenes* counts. The MacConkey and Oxford plates were incubated at 37°C, over night and 48 to 72 h, respectively. In order to assess the selective medium recovery capacity, one of the experiments in pure culture for each species was performed in the corresponding selective medium and in TSA. No significant differences were found between the CFU counts in TSA and in the two selective media (data not shown).

### Biomass quantification by the CV Assay

Quantification of biofilm production was based on the previously described method [35]. The washed coupons were placed in a new six-well tissue culture plate and fixed with 3 ml of methanol 98% (vol/vol) for 15 m. Following, the methanol was removed and the coupons were allowed to air-dry. Then biofilms were stained with 3 ml of CV (Merck) for 5 min. Coupons were washed three times, by pouring tap water over the coupon, allowed to air-dry, and then the CV was removed by adding 6 ml of 33% (vol/vol) glacial acetic acid (Merck) to each well. The plates were placed in agitation for a few minutes and 250 µl were transferred to a 96-well microtiter plate. Subsequently the OD was measured at 570 nm using a microtiter plate reader (Model Sunrise, Tecan).

### PNA FISH hybridization and DAPI staining

A specific 23S rRNA PNA probe (SalPNA1873) previously developed [36], was used for *S. enterica* detection. For *L. monocytogenes* detection, we searched for conserved regions in the rRNA sequences using the ClustalW [37]. The alignments showed a *L. monocytogenes* conserved region for the 16S rRNA. Based on the GC percentage, presence or absence of self-complementary structures and melting temperature similar to the one of the *Salmonella* probe, the following PNA oligomer sequence was selected: 5′-GAC CCT TTG TAC TAT -3′. This sequence hybridizes between the position 1253 and 1267 on *L. monocytogenes* EGD-e 16S rRNA sequence (accession number NC_003210) (Figure S1). The probe was designated LmPNA1253 due to the starting position on type strain EGD-e. The probe was synthesized (Panagene), attached to the Alexa 488 fluorochrome, and tested with *L. monocytogenes* ATCC 15313.

For biofilm samples cell quantification, a sonication step was included as described above and the PNA FISH hybridization was performed as previously reported in Almeida *et al.*, 2010 [36]. These PNA FISH counts were performed *ex situ* to have homogeneous counts and also because some materials, such as PVC and silicone, presented a strong autofluorescence signal, while for metal surfaces, steel and copper, the hybridization directly in the coupons did not work. Briefly, 1 ml of the sonicated samples for 24 and 48 h biofilm was pelleted by centrifugation at 10,000 g for 5 minutes, resuspended in 500 µl of 4% (w/v) paraformaldehyde and fixed for 1 h. The fixed cells were rinsed in autoclaved water, resuspended in 500 µl of 50% (vol/vol) ethanol and incubated for 30 min at −20°C. Subsequently, 100 µl of the fixed cells aliquot was pelleted by centrifugation, rinsed with sterile water and resuspended in 100 µl of hybridization solution containing 10% (wt/vol) dextran sulphate, 10 mM NaCl, 30% (vol/vol) formamide, 0.1% (wt/vol) sodium pyrophosphate, 0.2% (wt/vol) polyvinylpyrrolidone, 0.2% (wt/vol) Ficol, 5 mM disodium EDTA (Sigma), 0.1% (vol/vol) Triton X-100, 50 mM Tris-HCl (pH 7.5) with 200 nM of PNA probe. The samples were incubated at 57°C for 30 min. After hybridization, cells were centrifuged at 10,000 g for 5 min, resuspended in 500 µl of wash solution containing 5 mM Tris Base, 15 mM NaCl and 1% (vol/vol) Triton X (pH 10), and incubated at 57°C for 30 min. Washed suspension was pelleted by centrifugation and resuspended in 1 ml of sterile water. 200 µl of the cell suspension were filtered through a 25 mm black Nuclepore polycarbonate membrane with a pore size of 0.2 µm (Whatman, Kent, UK). As no PNA probe was used for *E. coli* detection, for biofilm samples containing this bacterium, the polycarbonate membrane were stained with 60 µl of 4′-6-Diamidino-2-phenylindole (DAPI; 100 µg/ml) for 10 m in the dark. Then the membrane was washed with 10 ml of water and placed in a microscope slide. Samples were allowed to air-dry, mounted with one drop of nonfluorescent immersion oil (Merck) and covered with coverslips. Cells were visualized under an epifluorescence microscope (BX51; Olympus) equipped with a CCD camera (DP71; Olympus) and filters capable of detecting the two PNA probes (BP 530–550, FT 570, LP 591 for SalPNA1873 and BP 470–490, FT 500, LP 516 for LmPNA1253) and DAPI (BP 365–370, FT 400, LP 421). A total of 10 fields with an area of 0,0158 µm^2^ were counted and the average was used to calculate the total cells per cm^2^. The experiment was performed in triplicate.

In other to assess the biofilm spatial organization and the species distribution, the PNA FISH and DAPI staining were also performed directly in the coupons. Before starting the hybridization, biofilm samples were dried at ∼60°C for 15 minutes and fixed with methanol (100%) for 10 minutes. This step showed to be very important to avoid the biofim detachment during the hybridization. After this, the PNA FISH procedure was identical to the one applied for slides in Almeida *et al.*, 2010 [36]. The DAPI procedure in coupons was performed as previously reported [34].

### Confocal laser scanning microscopy

The biofilm CSLM images were acquired in a FluoView FV1000 microscope (Olympus). Biofilms were observed using a 60× water-immersion objective (60×/1.2 W). Multichannel simulated fluorescence projection images and vertical cross sections through the biofilm were generated by using the FluoView application Software package (Olympus).

### Statistical analysis

Results were compared using One-Way analysis of variance (ANOVA) by applying Levene's test of homogeneity of variance and the Tukey multiple-comparisons test, using SPSS software (SPSS - Statistical Package for the Social Sciences, Chicago, USA) or Microsoft Office Excel (Microsoft Corporation, Redmond, CA). All tests were performed with a confidence level of 95%.

## Results

### PNA FISH method development and validation

The three microorganisms selected here as a case-study were *S. enterica*, *E. coli* and *L. monocytogenes*. *E. coli* is an indicator of the sanitary quality of the food-processing environment, and several *E. coli* strains have shown a great ability to produce biofilms on different surfaces [38]. *L. monocytogenes* and *S. enterica* are Gram-positive and negative bacteria, respectively, and are also important foodborne pathogens that persist on food contact surfaces due to biofilm formation [39], [40], [41]. As such, these pathogens present different cell wall properties and may share the same niches and persist in natural polymicrobial biofilms.

A probe for the detection of *Salmonella* spp. had already been developed by our group [36]. In here, a novel PNA FISH probe was developed for *L. monocytogenes*, targeting the positions 1253 to 1267 of the 16S rRNA *L. monocytogenes* strain EDG-e (accession number: AL591974.1) (Figure S1). Because the intention was to form mono-, dual- and tri-species biofilms, both probes would have to be specific to the species of interest at the same hybridization and washing temperature in a multiplex experiment. As most naturally-occurring mixed biofilms will consist of more than three species, we have decided to use 4′,6-diamidino-2-phenylindole (DAPI) as a non-specific nucleic acid dye to detect all cells that are present, here represented by *E. coli*. The selection of this *E. coli* strain to represent all other species was based on the fact that, in preliminary experiments, this microorganism always outnumbered the other two species in biofilms.

**Figure 1:**
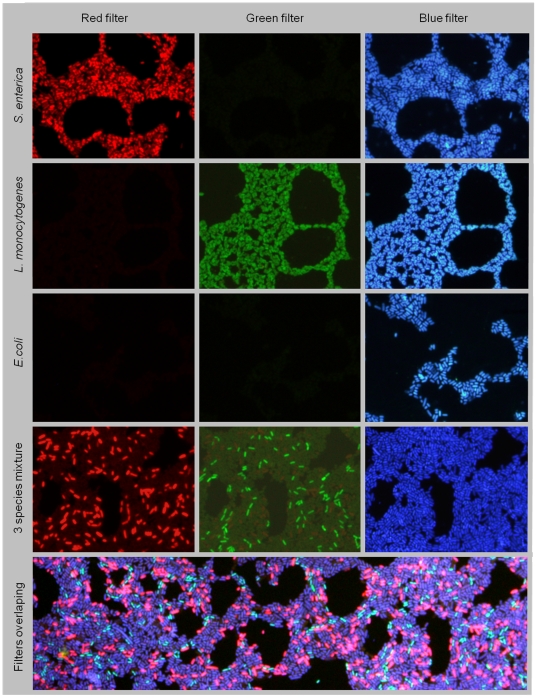
Epifluorescence microscopy pictures of a multiplex assay for mono-species and a three-species smear, using two PNA probes (SalPNA1873 and LmPNA1253) and DAPI staining. In the columns we have the microscopy filter used to visualize each fluorochrome (from left to right, Alexa 594, Alexa 488 and DAPI). The first three rows present the pure smears for each species used. No cross-hybridization was observed between the two PNA probes. The fourth row shows a smear with the three species mixed. The bottom image represents the bands superposition discriminating the cells of the three populations.

The hybridization conditions were optimized for the target microorganism and the probes specificity was evaluated on pure cultures. For this, a mix of the two probes was prepared and applied to *E. coli*, *S. enterica* and *L. monocytogenes* pure culture smears. After the PNA FISH hybridization the samples were also stained with DAPI. Microscopic visualization showed that both PNA probes provided a strong fluorescent signal at 57°C, and no cross-hybridization was observed between the probes (Figure 1, rows 1 to 3). Next, the method was tested with a smear of the three species mixed together and results showed that it provided an accurate discrimination between the three species involved (Figure 1, fourth and fifth row). A red background on the green channel is slightly visible due to a small overlapping between the Alexa 594 absorption spectrum and the excitation filter (band pass [BP] from 470 to 490) used to visualize the LmPNA1253 probe. As such, and because the green barrier filters is a long pass filter (LP 516), the Alexa 594 fluorescence emission is still detected.

### Quantification of biofilm populations by PNA FISH

**Figure 2:**
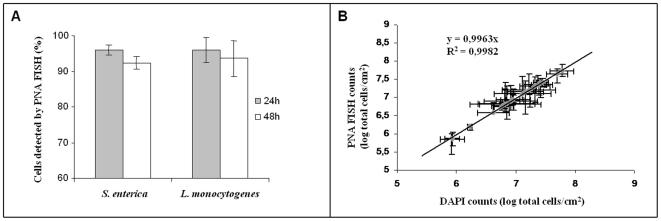
PNA FISH validation for biofilm samples. (A) Percentage of cells detected by PNA FISH for 24 and 48 h biofilms, in comparison with the total cells counts by DAPI. (B) Correlation between the PNA FISH counts and the DAPI counts for 24 and 48 h *S. enterica* e *L. monocytogenes* pure- culture biofilms. A high correlation between the two methods was observed and up to 48 hours at least 90% of the populations is detected by PNA FISH.

After testing the multiplex assay on smears, our first aim was to establish the limits of the PNA FISH method for a quantitative assessment of cells in a membrane. Initially, we assessed by DAPI staining and fluorescence microscopy that sonication in these conditions removed more than 99% of the biofilm cells for all materials and species. Subsequently, we compared the results obtained by PNA FISH at both 24 and 48 h with those obtained from DAPI for the *S. enterica* and *L. monocytogenes* pure cultures biofilms on the seven materials. As expected, the differences between PNA FISH and DAPI counts increased with biofilm formation time (see Figure 2AFor instance, for *S. enterica* biofilms the percentages of cells detected by PNA FISH were 95,8% (±1,7) for the 24 h, 91,4% (±1,8) for 48 h and decreased for 56,1% (±4,1) for 192 h. Nevertheless, up to 48 hours, PNA FISH counts always represented more than 90% of DAPI counts (Figure 2A), and this correlation was independent of the species, number of the cells and the type of support material used for cell adhesion (slope = 0.9963; R = 0.9982) (Figure 2B).

As the numbers of undetected PNA FISH cells automatically add up to the numbers of cells considered to be *E. coli*, it is important to bear in mind that this strategy is only reliable if the population that is identified indirectly by DAPI outnumbers the populations quantified by PNA FISH, which is the case here. If this is not the case, for biofilms containing more than three species it may be possible to use universal or group-specific probes to detect all other microbial species, such as the EUB 338/BacUni-1 [42], [43], [44]. However, it was previously shown that to more accurately detect all biofilm bacterial population, at least three probes should be combined, which can greatly increase the complexity of the hybridization [45].

### Single and dual-species biofilm experiments

**Figure 3:**
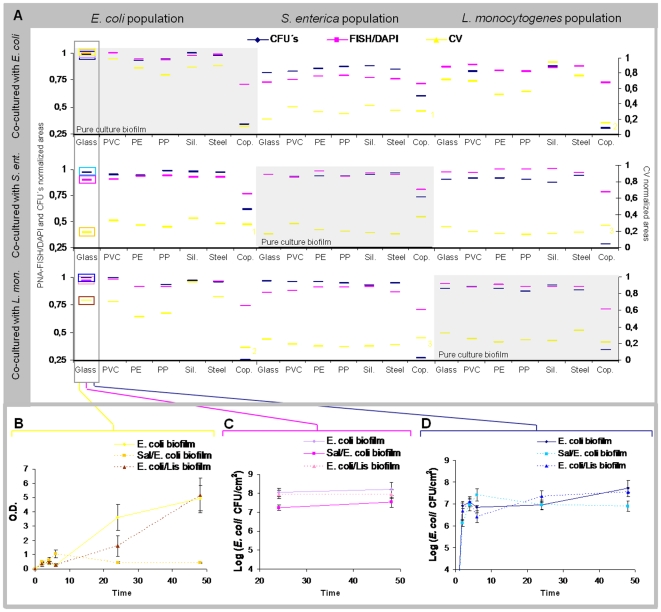
Biofilm formation for single- and dual-species biofilms. On panel A it is possible observe the normalized areas for each biofilm on each adhesion material for cultivability, CV and PNA FISH/DAPI graphs (A). Panels B, C and D are shown as examples of CV, PNA FISH/DAPI and cultivability graphs, respectively, on the glass support. Similar graphs for the remaining supports are provided in the Figures S2, S3, S4 and S5.

Taking advantage of the robustness of the PNA FISH/DAPI method, we next investigated the strains ability to form biofilms in 7 different materials, and complemented the obtained results with two other well-known analytical methods: CFU counts and CV. This allows to determine how the behavior of each strain is affected in the presence of a different species in terms of cultivability, biofilm biomass and individual cell counts. A total of six biofilm experiments (three single-species and three dual-species biofilms - *E. col*i/*S. enterica*; *E. coli*/*L. monocytogenes* and *S. enterica*/*L. monocytogenes*) were performed in triplicate (Figures S2, S3, S4 and S5). Due to the large number of data obtained with the 3 methods, we determined the area under the cultivability, CV and PNA FISH curves by the trapezium rule, as described previously [46](Figure 3.A). Three areas were obtained for each method and biofilm experiment as exemplified in Figures 3B, C and D only for the glass support, from which the final average area was calculated. In order to normalized the areas, each value was divided for the higher value on each data serie, for instance, all CV areas were divided for the higher CV area, which was obtained for *E. coli* biofilm on glass. Results show that biofilm patterns are quite similar for six of the materials tested, regardless of the analytical method used (P>0,05 in all cases) (Figure 3.A). The exception was copper, which presented an inhibitory effect on biofilm formation for all three species (P<0,05).

For the conditions at which the experiments were carried out (21°C on TSB), the growth rate of *E. coli* and *Salmonella*, was found to be similar (approx. 0,104 h^−1^). However, all analytical methods showed that *E. coli* was the best biofilm producer (P<0,05) (Figure 3 and Figure S2). It is also important to notice the *E. coli* capacity to produce a great amount of exopolymers, easily visible to the naked eye in all the materials used, except for copper. This characteristic is reflected in the CV data for pure culture, where we can observe high CV areas for *E. coli* biofilm, which reached an optical density of ∼5 for 48 h (Figure 3.B) and cell densities of ∼Log 7.5 (Figure 3.D). The other two species presented lower ability to form biofilm comparing to *E. coli*, but were comparable between each other. Despite the lower growth rate of *Listeria* (0,088 h^−1^), both *S. enterica* and *L. monocytogenes* pure culture biofilms presented similar values for both total biomass (DO∼2 for 48 h) and cultivability (∼Log 6.5–7 for 48 h) (Figure S2).

**Figure 4:**
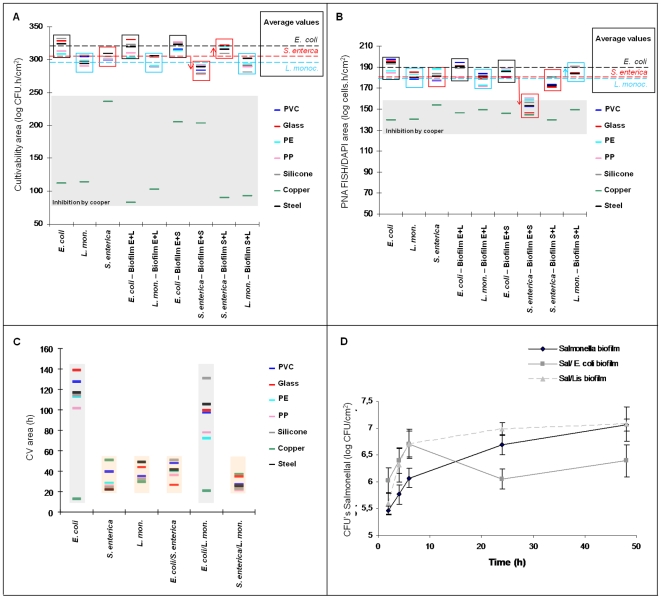
Biofilm formation profiles for each species on single- and dual-species biofilms. Cultivability (A) and PNA FISH/DAPI (B) areas showing the populations variations when co-cultured with a different species. (C) CV areas showing two typical CV profiles, the *E. coli* profile (at grey) suggesting a high production of exopolymers, and the *L. monocytogenes* and *S. enterica* profile (at pink) showing a reduced ability to produce exoplimers. The CV profile for *E. coli/S. enterica* biofilm suggests that *Salmonella* affected the *E. coli* ability to produce exopolymers.

To better visualize eventual species-specific profiles on different materials and check for possible species interactions, we grouped together the areas according to the microorganism(s) that formed the biofilm (Figure 4). The type of adhesion material was not a major determinant on the amount of biofilm produced (except for copper); in fact, the major factor appears to be the microorganism(s) that form the biofilm as the 3 species maintained the same profile for 6 of the materials.

In terms of strains interactions in dual-species biofilms, in general, cell densities of *E. coli* and *L. monocytogenes* were not affected by the presence of other species (Figure 4 A e B). On the other hand, the numbers of *S. enterica* in the biofilm decreased in the presence of *E. coli*, probably due to the competition for nutrients, and *S. enterica* numbers increased slightly in terms of cultivability areas (Figure 4.A) when co-cultured with *L. monocytogenes*. For *S. enterica*, total cell number appeared to be more affected than cultivability. However, when plotting the *Salmonella* cultivability averages for all materials (except copper) with time (Figure 4.D), it was observed that this difference is mainly due to an increased initial adhesion of *Salmonella* when co-cultured,and as PNA FISH values only comprise the last 24 h, the PNA FISH areas are comparatively smaller (Figure 4.B). Regarding *Listeria*, as it is a slow-growing bacterium [47], probably there is less competition for nutrients and the presence of *Listeria* was unaltered either when co-cultured with *E. coli* or with *S. enterica*. For PNA FISH method, it was even observed a small increase on *Listeria* population, which was not visible on cultivability data.

Regarding copper influence, it was observed that *S. enterica* maintains the resistance to copper when co-cultured with *E. coli* and also seems to improve the survival of *E. coli* on cooper (Figure 4). On the other hand, when co-cultured with *Listeria*, *Salmonella* seems to be less resistant to copper, while *Listeria* cultivability remains similar.

Comparing the dual-species biofilm CV areas with those for a single-species (Figure 4.C), it can be observed that *Listeria* and *Salmonella* mixed biofilm maintained the same biomass profile. When *Listeria* is co-cultured with *E. coli*, the profile observed in the CV areas is similar to the one for *E. coli* biofilm. This may happen because, as we said before, this *E. coli* strain produce a great amount of exopolymers and, as *Listeria* is a slow-growing bacterium, they are not competing for nutrients and then the CV profile is characteristic of *E. coli*. On the other hand, when *E. coli* is co-cultured with *S. enterica*, the CV area profile is similar to the *S. enterica* biofilm, which means that probably *S. enterica* is affecting the *E. coli* ability to produce exopolymers. Even though, *E. coli* remains in higher numbers (Figure 4.A and B).

**Figure 5:**
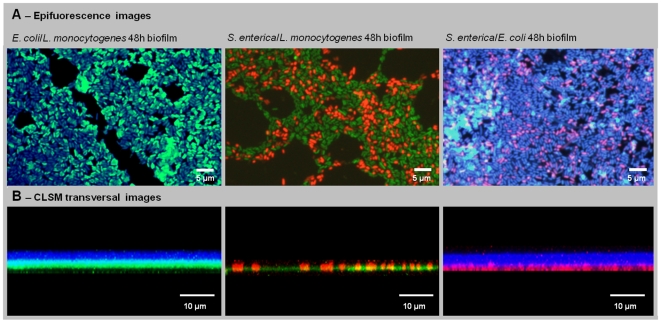
Dual-species biofilms spatial organization for 48 h. (A) Epifluorescence images showing an homogeneous distribution of the species. (B) CLSM transversal images showing that dual-species biofilms with *E. coli* presented two well defined layers. For *Salmonella*/*Listeria* biofim, it was not observed the formation of two layers.

Regarding spatial distribution, epifluorescence microscopy showed that on the XY axys the species were equally distributed across the surfaces of all 3 dual-species experiments (Figure. 5A). Nevertheless, observation under CLSM (Figure. 5B), demonstrated that dual-species biofilms with *E. coli* presented two well defined layers in the Z-axys. The first, close to the material surface, with *S. enterica* or *L. monocytogenes*, and another layer mainly constituted of *E. coli* cells. For *Salmonella*/*Listeria* biofim, it was not observed any obvious preferential vertical distribution. We also did not see the formation of differentiated three-dimensional structures like “mushrooms” or “stacks” of microcolonies [48].

### Comparison between cultivability and PNA FISH/DAPI

**Figure 6:**
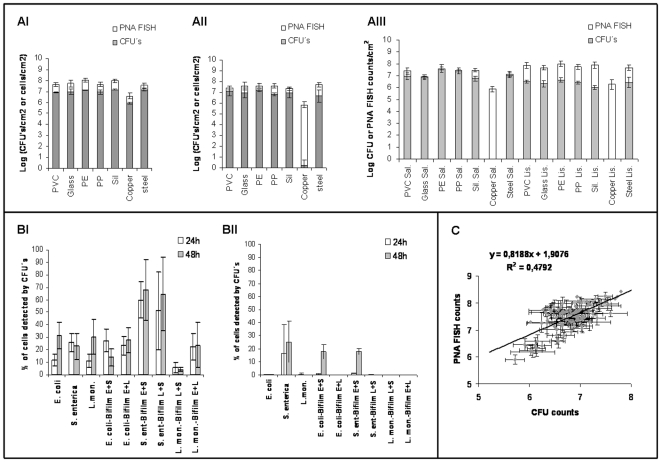
Comparison between PNA FISH/DAPI and cultivability measurements. Viable and cultivable bacteria adhered to the different material for *S. enterica* (AI) and *L. monocytogenes* (AII) pure culture biofilm and *Salmonella/Listeria* dual-especie biofilm (AII). Percentages of cells detected by cultivability for each specie, on single and dual-specie biofilm, adhered to copper (BII) and the remaining six material (BI- average values determined for the six materials together). Correlation between the PNA FISH counts and the CFU counts for 24 and 48 h biofilms (C) (all the 6 biofilm experiments included).

For 24 and 48 h, and as expected, it was observed that the CFU values were always lower than the FISH/DAPI values, no matter the type of support material, the strain (Figure 6.AI and AII) or the presence of a co-cultured strain (Figure 6. AIII). However, when compared with the other materials, it was observed an accentuated loss of cultivability for biofilms adhered to copper (Table S1).

Regarding the remaining materials, its properties do not seem to have a great influence in the cultivable state of the cells. As so, we determined the average of cells detected by cultivability on each biofilm for the 6 materials (Figure 6.BI) and comparison showed that these values were much higher than those obtained for copper (Figure 6.BII).

It was also observed an increase on the *Salmonella* cultivability when co-cultured with both *E. coli* and *Listeria* (Figure 6.BI). To determine whether there was a correlation between cultivability and PNA FISH/DAPI numbers, we also plotted the 24 h and 48 h cultivability values with PNA FISH/DAPI counts for the 6 biofilm experiments. We found a weak correlation between the two methods (slope = 0.8188; R = 0.4792) (Figure 6.C), probably due to differences on how the physiological state of different bacteria is affected by the presence in biofilms, other microorganisms and support material.

### Three species-biofilm

As a final experiment, we characterized a mixed biofilm of *E. coli*, *L. monocytogenes* and *S. enterica* using PNA FISH and DAPI staining combined with CLSM., in terms of each constituent population, spatial organization and cell numbers. This information allowed us to infer about inter-species interactions of a three-species mixed-biofilm.

**Figure 7:**
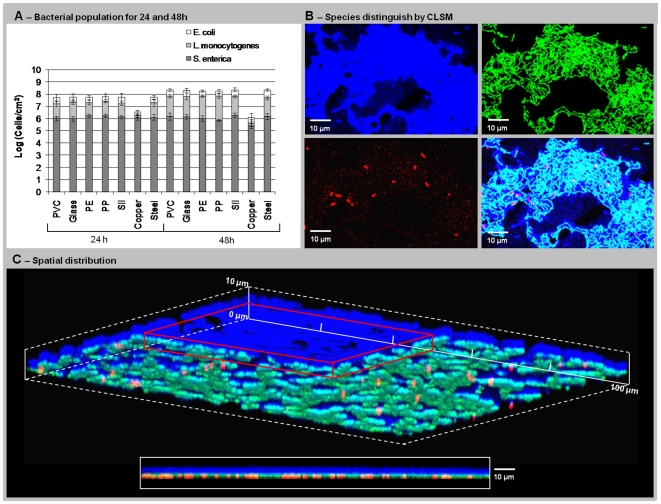
Tri-species biofilm formation. (A) Biofilm populations for 24 and 48 hours on each support material. (B) CLSM images distinguishing each bacteria and the superposition of the three fields. (D) CLSM showing the biofilm three-dimensional spatial distribution. A frontal quadrant (red rectangle) was removed to show the existence of an upper layer exclusively formed by *E. coli,* over a mixed *Salmonella* and *Listeria* layer. The bottom blank rectangle shows a transversal biofilm image showing the well defined layers.

Results showed that *Salmonella* and *Listeria* species initial adhesion (2 h) is similar (Log 4.5 to 5.5 CFU/cm^2^), while the number of *E. coli* adhered for a 2 h biofilm is slightly higher (Log 5 to 6 CFU/cm^2^) (Figure S6). For 24 and 48 h, *E. coli* remained the outnumbered population [2,1×10^8^ cells/cm^2^ (±2,4×10^7^)], followed by *Listeria* [6,8×10^7^ cells/cm^2^ (±9,4×10^6^)] and *S. enterica* [1,4×10^6^ cells/cm^2^ (±4,1×10^5^)], and this last one was clearly inhibited by the other species (See Figure 7.A). For copper these proportions were similar, but cellular densities were lower: *E. coli* 1,5×10^6^ cells/cm^2^ (±8,9×10^5^), *Listeria* 5,4×10^5^ cells/cm^2^ (±2,7×10^5^) and *S. enterica* 2,8×10^5^ cells/cm^2^ (±1,2×10^5^). Regarding spatial structure, an extension of the results obtained for two-species biofilms could be observed using the two PNA probes simultaneously counterstained with DAPI (Figure 7.B). As before, an *E. coli* layer of ∼3 to 4 µm can be clearly identified on top of the biofilm, whereas a thinner layer (∼2 to 3 µm) of *S. enterica* interspersed with *L. monocytogenes* could be observed next to the surface of the materials (Figure 7C).

## Discussion

### PNA FISH discrimination and quantification of populations in biofilms

One of the major hurdles in multispecies biofilm research is to correctly quantify and locate each of the species present. Up until now, there was a lack of a suitable method that could both quantify and locate microorganisms in a biofilm in a reliable way. In fact, the few studies that have previously attempted to quantify different populations in a biofilm [49], [50], [51], [52], were either unable to provide absolute cell numbers of populations in biofilms (and estimated percentages of populations instead), or used quantitative PCR as a complementary technique to perform that task.

In this work we evaluated the adaptability of PNA-FISH counterstained with DAPI to simultaneously quantify and locate three microorganisms in biofilms. Tests on pure culture smears showed that this method is able to distinguish 3 different microorganisms with distinct cell wall characteristics in a multiplex experiment. While detecting both Gram- and Gram+ microorganisms using the same fixation/permeabilization method has been proved difficult for conventional DNA probes [23], the particular characteristics of PNA (such as the uncharged backbone), had already allowed to develop a multiplex experiment for both groups of microorganisms as early as 2001 [44]. The uncharged nature of PNA was arguably one of the main determinants in allowing the detection of microorganisms from the basal to the top layer of a 7 µm thick biofilm. The presence of charged particles in the biofilm matrix, such as DNA and sugar-acid residues [53], could act as repulsors to the passage of DNA probes but had no observable effect in our experiment.

Despite the advantages of PNA FISH, so far we were only able to confidently quantify biofilms populations ex-situ and up to 48 hours. This was somewhat expected, as the PNA FISH signal is based in rRNA content and as the biofilm cells become dormant, a reduction of the rRNA content occurs [54], [55], [56], [57]. Recently, minimal numbers of rRNA copies per cell of *E. coli* needed to obtain a visible signal of a microbial cell after FISH have been estimated to be in the order of 370 molecules for cells hybridized on glass slides [58]. With future advances on both fluorochrome design and microscopy [59], [60], [61], it is however expected that this threshold will decrease with time.

### Biofilm experiments

As a case study, biofilm experiments were performed to evaluate the ability of *S. enterica*, *L. monocytogenes* and *E. coli* to form pure and mixed-species biofilms in seven different support materials and with time.

Due to the large amount of results obtained, data were presented as areas instead of the original graphics of cultivability/total cell counts/exopolyssacharide production over time. We are aware that by presenting data in this way, some information is lost, such has the kinetics of cell adhesion and the absolute values for the three assessed parameters at the pseudo-steady state. As such, the original graphics should always appear as supplementary information when this strategy is used.

All strains maintained the biofilm profile for 6 of the materials suggesting that biofilm formation was mainly controlled by species-associated phenomena. The exception was copper, that clearly inhibited biofilm formation for all species regarding both expolymer production and number of cells adhered, and also seems to promote the shift to the viable but noncultivable (VBNC) stage, as the percentage of cells not-detected by cultivability is much higher for biofilms adhered to copper. This toxic effect of copper, had already been reported by other authors for different bacteria, and is probably due to the Cu^2+^ ions release to the medium, causing for example degradation of genomic and plasmid DNA and inhibition of respiratory activity [62], [63], [64], [65]. Other authors have already showed that copper ions may be used in combination with some biocides to eradicate biofilms of *E. coli*, *Staphylococcus aureus*, *S. enterica* and other species [66]. Our result show that exposure to copper is still deleterious even for mixed species biofilms, and copper ions appear to act equally on all three populations present making this material particularly suitable for inhibiting biofilm growth.

In this study, *E. coli* was the best biofilm producer in single, dual and tri-species biofilms, and always outnumbered the remaining populations. The exceptional capacity to adhere and form an abundant matrix in a broad range of materials by our *E. coli* ATCC 25922 had already been observed [67]. *Listeria* seems to deal better with competition on mixed biofilm, while *Salmonella* population decreases when co-cultured with *E. coli*. On the other hand, despite *Listeria* low growth rate and limited ability to form biofilm (comparing to *E. coli*), it seems to adapt very well to mixed biofilms. This behavior was already reported by other authors [68], [69], who have shown that *Listeria* can either exist in monoculture biofilms or be a part of mixed culture biofilms with bacteria such as *Flavobacterium* and *Comamonas testosterone*, both Gram- bacteria. It even increased the number of cells attached to the surface and this cells could survive for a longer period of time in a mixed culture biofilm[68].

### Three-species biofilm development model

**Figure 8:**
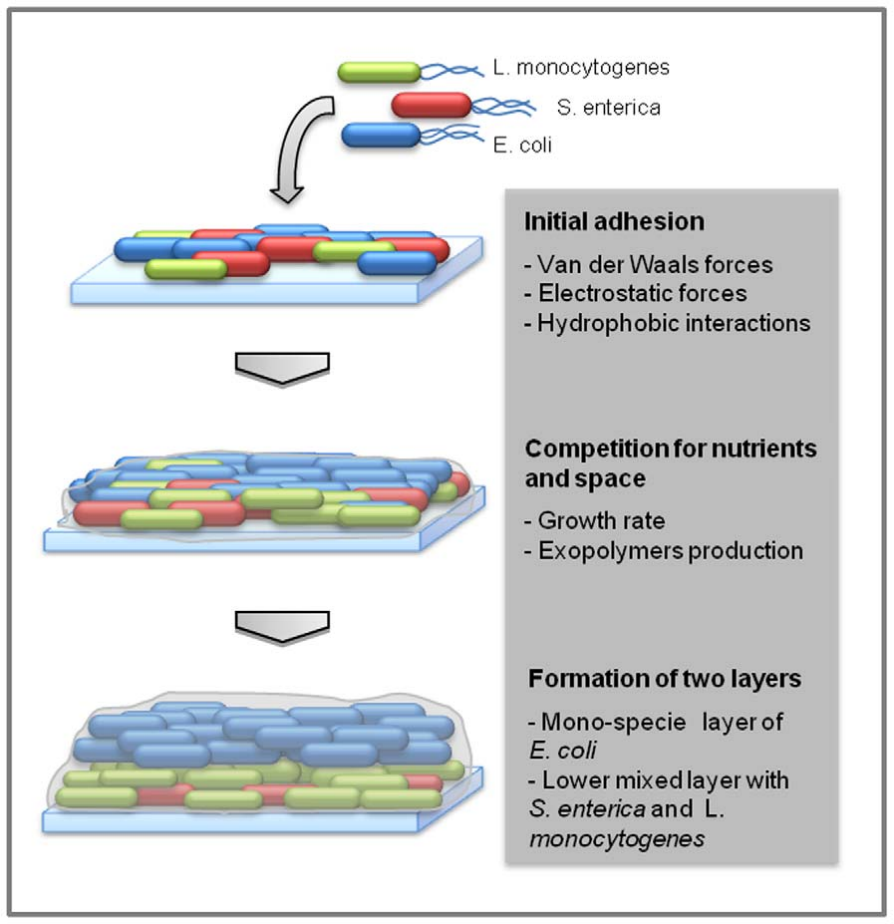
Schematic representation of the tri-species biofilm formation showing the main steps and the key factors involved on the two layers appearing.

By combining widely accepted views of biofilm formation and the results obtained in this work, we were able to reach a final development model for our three-species model biofilm (Figure 8). Initial adhesion involves cell proximity to the surface to allow for reversible or irreversible attachment, which is governed by van der Waals forces, electrostatic forces and hydrophobic interactions [70], [71], [48]. For this three species biofilm this initial adhesion is slightly higher for *E. coli* (Figure S6), which together with the high growth rate (when compared to *Listeria*) and exopolymer production, probably allows *E. coli* to proliferate, while keeping other bacteria on the bottom of the biofilm. Despite the similar growth rate to *E. coli* when in pure culture, *S. enterica* was not able to proliferate in the three-species biofilm perhaps due to the *E. coli* presence, which was evidently harmful for *Salmonella* in the dual-species biofilm. This antagonistic effect is probably caused by exopolymer production by *E. coli*. In fact, as the matrix involves the remaining species it probably hinders *S. enterica* and *Listeria* access to nutrients and oxygen, and consequently their growth. Diffusion limitations would also result in high concentration of CO_2_ [48], mostly released form *E. coli* metabolism. All these factors together: low available oxygen, high CO_2_ concentrations and limited access to nutrients, may explain the lower *S. enterica* and *L. monocytogenes* concentration on the biofilm (model described on Figure 8). Nevertheless, *L. monocytogenes* seems to deal better with these adverse conditions, and can proliferate and outnumber *S. enterica* population. But, as it grows slowly, it still stays at the bottom of the biofilm. *L. monocytogenes* ability to survive under adverse conditions was already reported by several authors who showed that it can adapt to and resist to different stress factors such as, low temperatures, starvation, high CO_2_ concentrations, presence of salts and organic acid and low pH [72].

Despite mixed biofilms being the most common biofilms in nature, there is a lack of studies trying to understand multispecies interactions within these structures. This is partly due to the need of reliable techniques for the quantification, visualization and discrimination of each population. This work establishes PNA FISH as a robust method to confidently discriminate multispecies biofilms and thus infer about multi-species interactions.

## Supporting Information

Figure S1 LmPNA1253 probe and it sequence target on L. monocytogenes EDG-e16S rRNA. The rRNA secondary structure was predicted using the RNAfold Program (http://rna.tbi.univie.ac.at/cgi-bin/RNAfold.cgi). The positional entropy reports to the energy of that position in space, which is related with the stability of that position. As we can see, the probe matches a region with an intermediate stability and, therefore, the access to that region should be easy.
(0.39 MB TIF)

Figure S2 Cultivability, cristal viotel and PNA-FISH/DAPI assays for single-specie biofilm experiments.
(0.17 MB TIF)

Figure S3 Cultivability, CV and PNA-FISH/DAPI assays for *E. coli*/*L. monocytogenes* dual-species biofilm.
(0.12 MB TIF)

Figure S4 Cultivability, CV and PNA-FISH/DAPI assays for *E. coli*/*S. enterica* dual-species biofilm.
(0.11 MB TIF)

Figure S5 Cultivability, CV and PNA-FISH/DAPI assays for *L.monocytogenes*/*S. enterica* dual-species biofilm.
(0.12 MB TIF)

Figure S6 Tri-species biofilm initial adhesion (2 h), for the seven materials used. *Salmonella* and *Listeria* presented similar initial adhesion. E. coli initial adhesion is slightly higher.
(0.07 MB TIF)

Table S1 Percentage of cells detected by cultivability method for 24 h and 48 h single and dual-specie biofilms, on each adhesion material, considering DAPI counts, for *E. coli*, and the PNA FISH counts, for *S. enterica*/*L. monocytogenes*, as the number of total bacteria.
(0.06 MB DOC)
