# Correction: Discriminating Multi-Species Populations in Biofilms with Peptide Nucleic Acid Fluorescence *In Situ* Hybridization (PNA FISH)

**DOI:** 10.1371/annotation/2b7e391f-b0cb-4d9f-8e90-85d080f8cbd4

**Published:** 2013-06-10

**Authors:** Carina Almeida, Nuno F. Azevedo, Sílvio Santos, Charles W. Keevil, Maria J. Vieira

There is a grant number missing from the Funding Statement. The following is the complete Funding Statement:

This work was funded by national funding of FCT (Portuguese Foundation for Science and Technology) and by FEDER through COMPETE (Programa Operacional Factores de Competitividade) under de project Micro2Micro - PTDC/EBB-EBI/104263/2008. C. Almeida also acknowledge FCT for the PhD Fellowship - SFRH/BD/29297/2006. The funders had no role in study design, data collection and analysis, decision to publish, or preparation of the manuscript. 

