# Supplementary figures and images for: Discriminating Multi-Species Populations in Biofilms with Peptide Nucleic Acid Fluorescence In Situ Hybridization (PNA FISH)
Source: PLoS One. 2011 Mar 29;6(3):e14786. doi: 10.1371/journal.pone.0014786 (PMC3066202; doi:10.1371/journal.pone.0014786)

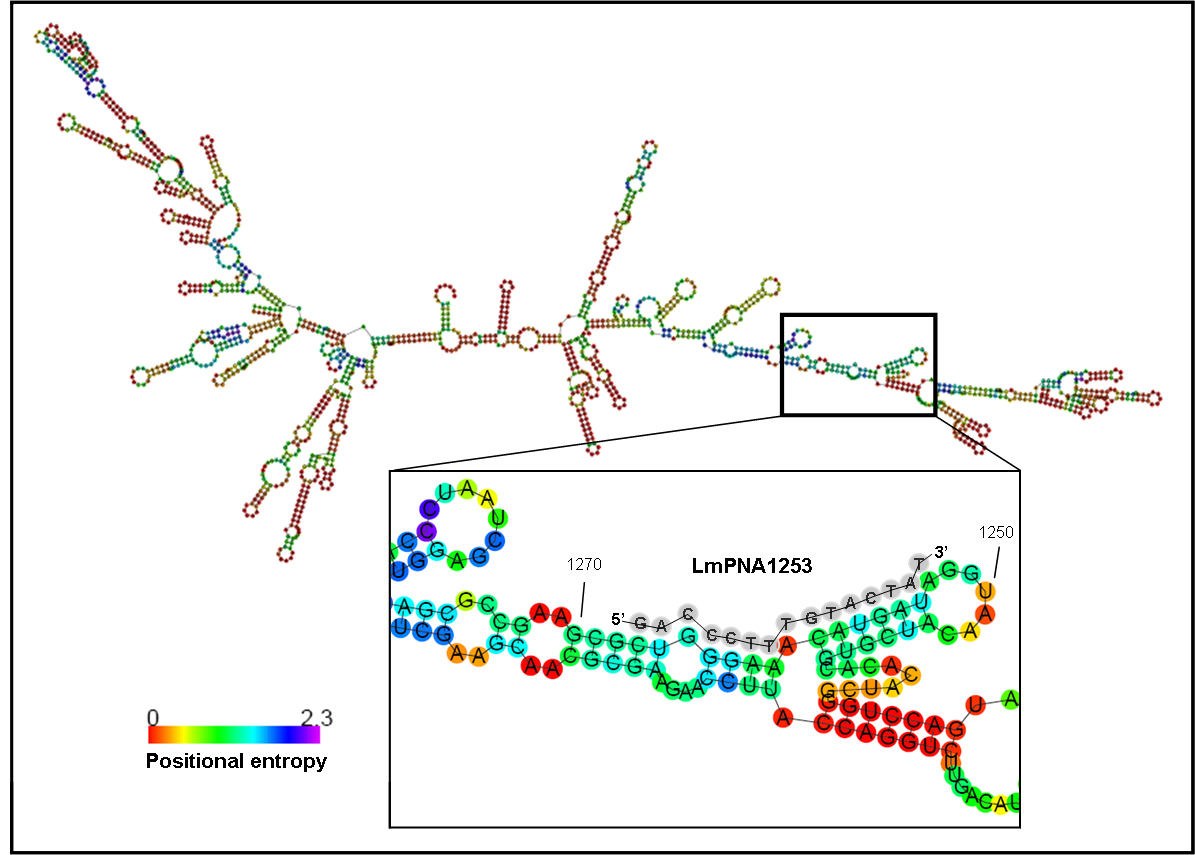

Supplement: Figure S1 — LmPNA1253 probe and it sequence target on L. monocytogenes EDG-e16S rRNA. The rRNA secondary structure was predicted using the RNAfold Program (http://rna.tbi.univie.ac.at/cgi-bin/RNAfold.cgi). The positional entropy reports to the energy of that position in space, which is related with the stability of that position. As we can see, the probe matches a region with an intermediate stability and, therefore, the access to that region should be easy. (0.39 MB TIF) [file pone.0014786.s002.tif]

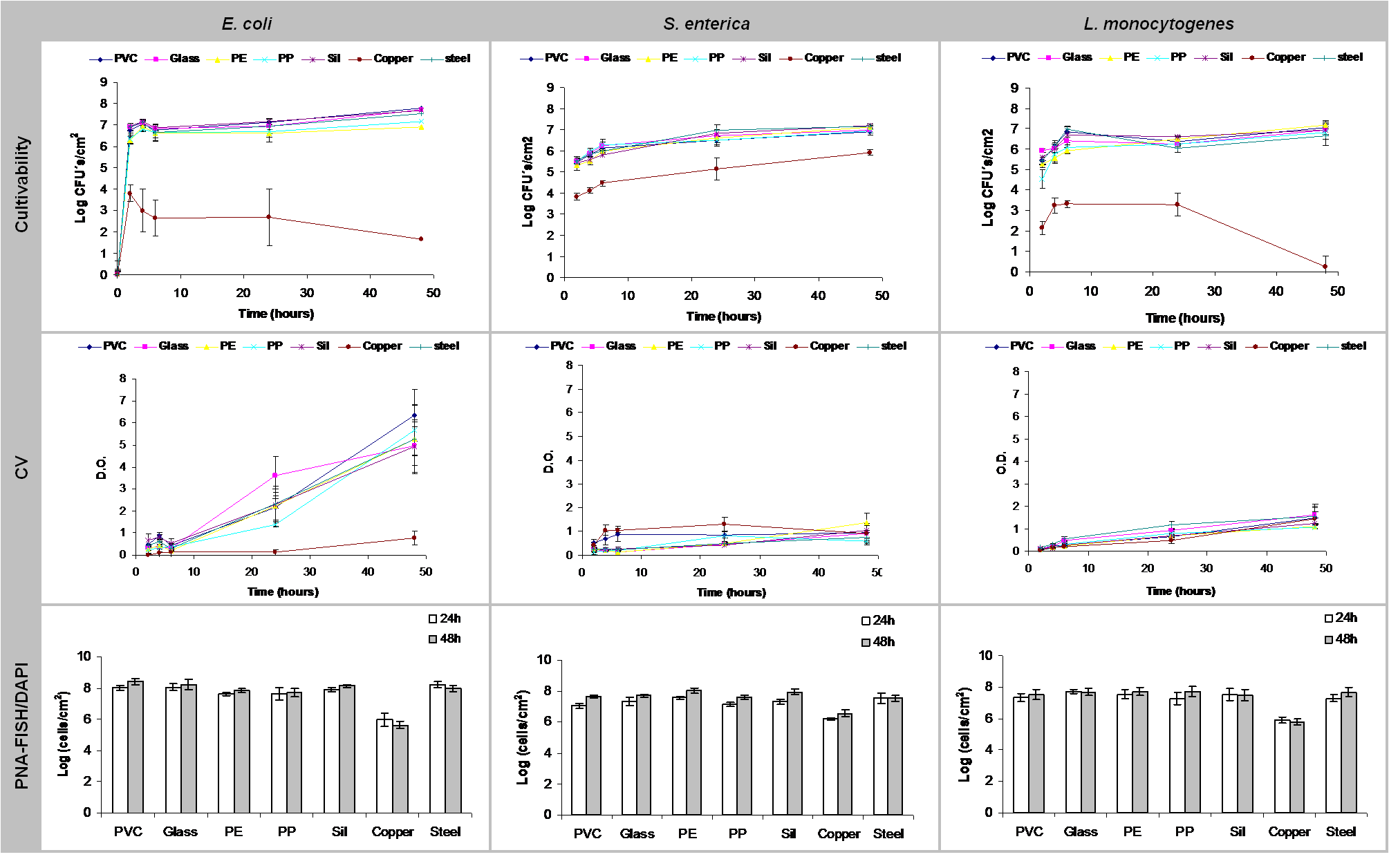

Supplement: Figure S2 — Cultivability, cristal viotel and PNA-FISH/DAPI assays for single-specie biofilm experiments. (0.17 MB TIF) [file pone.0014786.s003.tif]

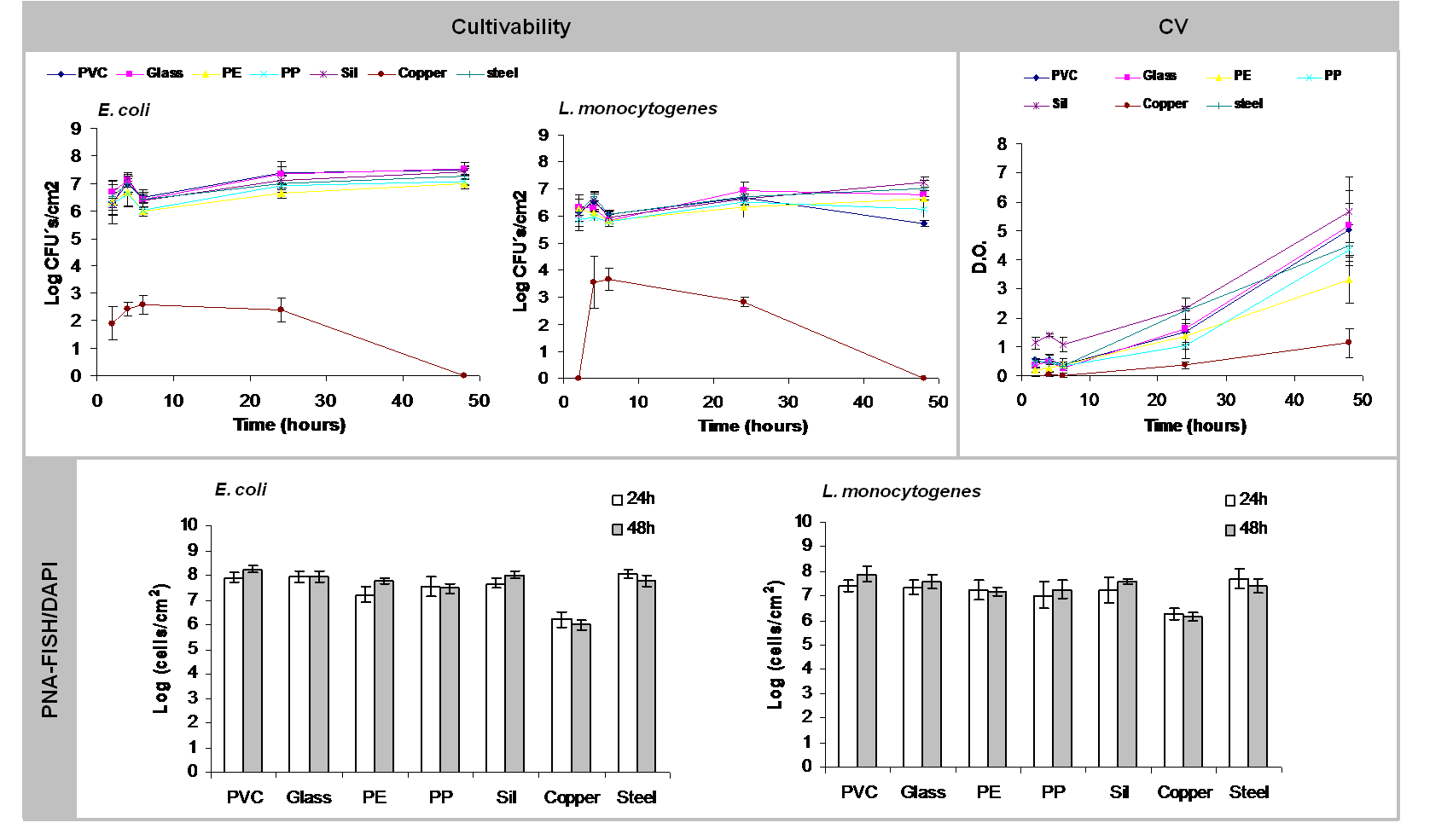

Supplement: Figure S3 — Cultivability, CV and PNA-FISH/DAPI assays for E. coli/L. monocytogenes dual-species biofilm. (0.12 MB TIF) [file pone.0014786.s004.tif]

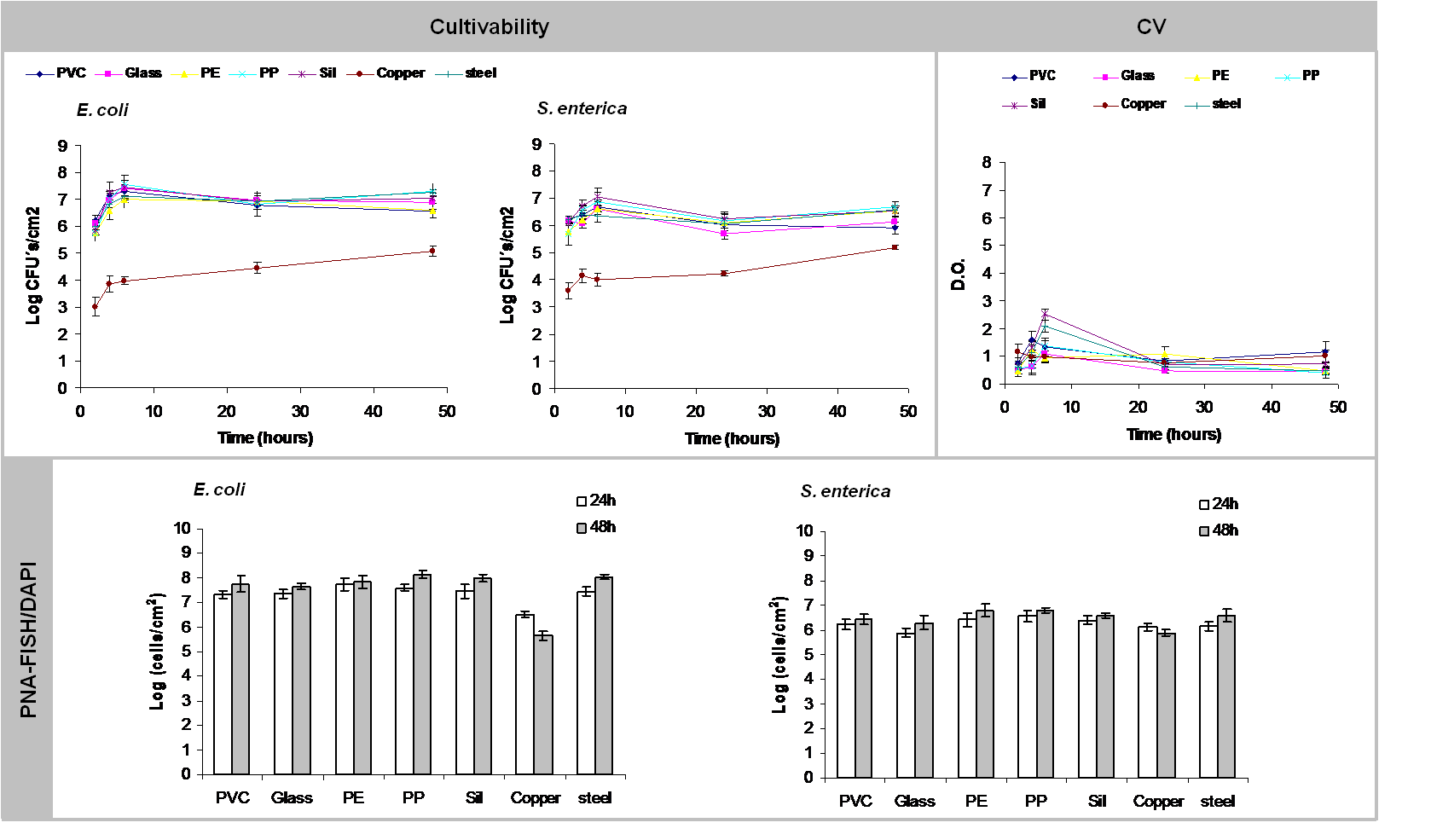

Supplement: Figure S4 — Cultivability, CV and PNA-FISH/DAPI assays for E. coli/S. enterica dual-species biofilm. (0.11 MB TIF) [file pone.0014786.s005.tif]

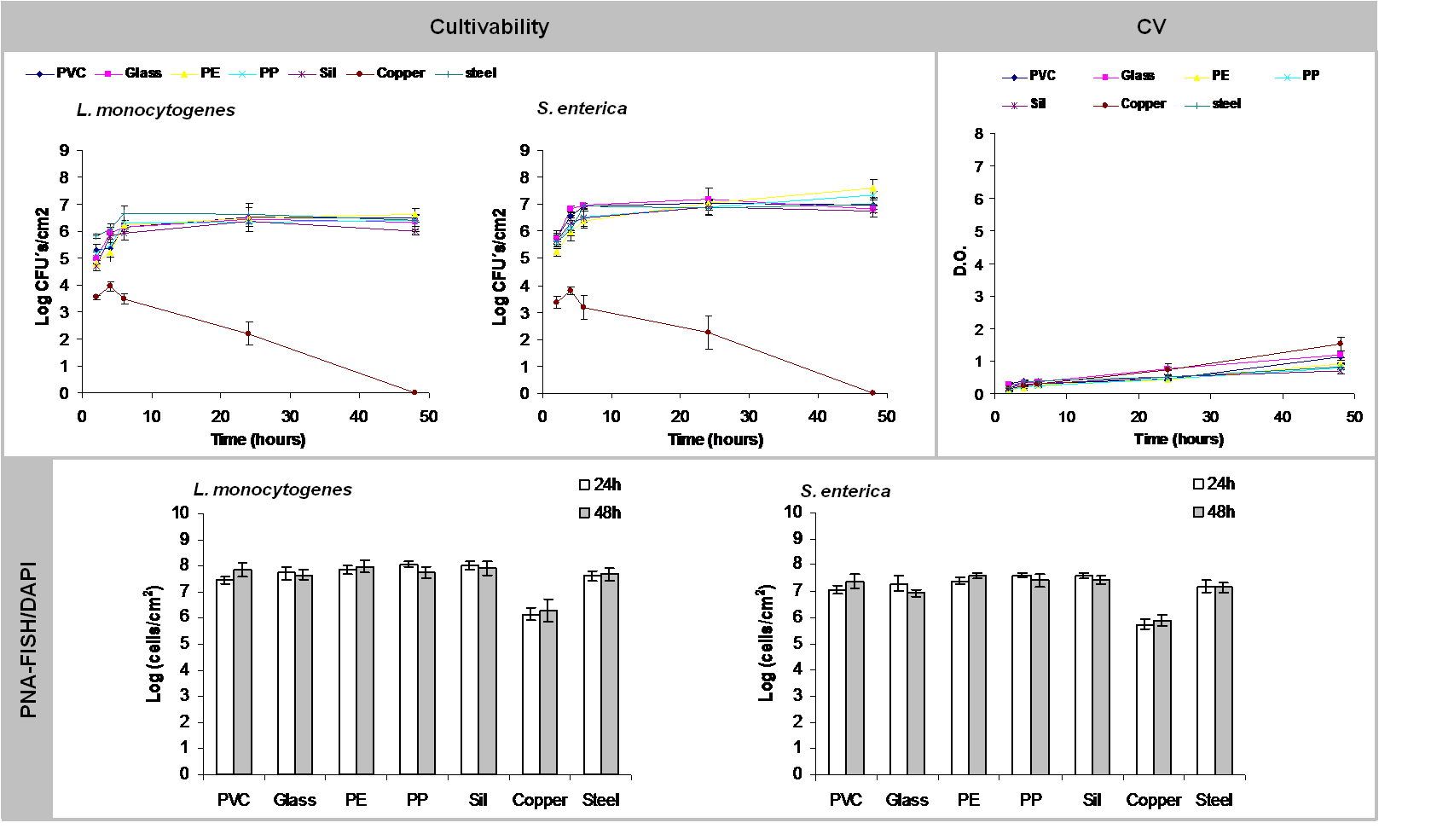

Supplement: Figure S5 — Cultivability, CV and PNA-FISH/DAPI assays for L.monocytogenes/S. enterica dual-species biofilm. (0.12 MB TIF) [file pone.0014786.s006.tif]

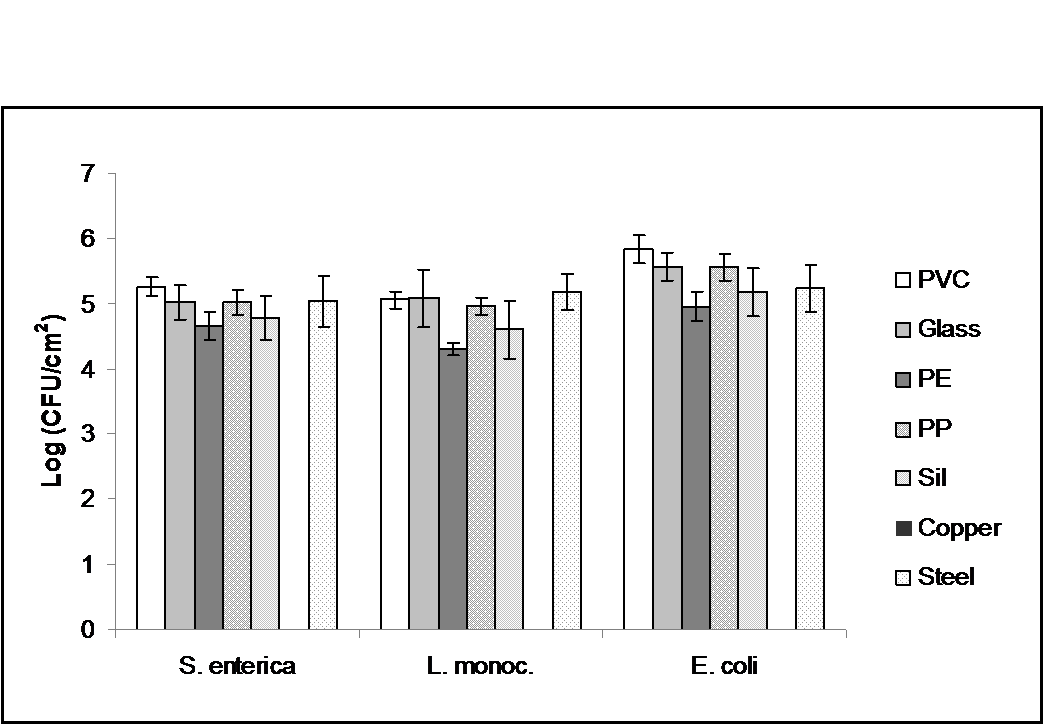

Supplement: Figure S6 — Tri-species biofilm initial adhesion (2 h), for the seven materials used. Salmonella and Listeria presented similar initial adhesion. E. coli initial adhesion is slightly higher. (0.07 MB TIF) [file pone.0014786.s007.tif]
