# Supplementary material for: Discriminating Multi-Species Populations in Biofilms with Peptide Nucleic Acid Fluorescence In Situ Hybridization (PNA FISH)
Source: PLoS One. 2011 Mar 29;6(3):e14786. doi: 10.1371/journal.pone.0014786 (PMC3066202; doi:10.1371/journal.pone.0014786)
Supplement: Table S1 — Percentage of cells detected by cultivability method for 24 h and 48 h single and dual-specie biofilms, on each adhesion material, considering DAPI counts, for E. coli, and the PNA FISH counts, for S. enterica/L. monocytogenes, as the number of total bacteria. (0.06 MB DOC) [file pone.0014786.s001.doc]

|  | | **% of cells detected** (± SD) | | | | | | |
| --- | --- | --- | --- | --- | --- | --- | --- | --- |
|  |  | **Glass** | **PVC** | **PE** | **PP** | **Silicone** | **Copper** | **Steel** |
| ***E. coli*** | 24h | 14,3  (± 5,7) | 8,4  (± 3,2) | 11,0  (± 7,0) | 12,2  (± 5,9) | 19,4  (± 11,5) | 0,29  (± 0,43) | 6,0  (± 4,4) |
|  | 48h | 24,5  (± 9,7) | 33,6  (± 17,0) | 13,4  (± 9,5) | 35,6  (± 22,9) | 40,8  (± 25,0) | 0,15  (± 0,24) | 39,7  (± 14,8) |
| ***S. enterica*** | 24h | 33,4  (± 22,7) | 55,4  (± 31,6) | 13,8  (± 10,4) | 25,2  (± 13,1) | 34,7  (± 12,1) | 16,4  (± 21,7) | 25,3  (± 0,05) |
|  | 48h | 19,9  (± 8,8) | 22,0  (± 15,8) | 13,0  (± 5,4) | 21,8  (± 10,6) | 17,2  (± 5,5) | 25,5  (± 15,1) | 41,9  (± 16,6) |
| ***L. mon..*** | 24h | 13,5  (± 7,7) | 3,5  (± 0,9) | 12,1  (± 7,8) | 15,1  (± 8,4) | 40,2  (± 34,3) | 0,6  (± 0,8) | 6,2  (± 1,9) |
|  | 48h | 43,3  (± 7,3) | 22,6  (± 3,8) | 36,3  (± 12,1) | 39,1  (± 31,4) | 40,7  (± 17,8) | 0,0004  (± 0,0006) | 10,8  (± 9,2) |
| ***E. coli***  **Bifilm E+S** | 24h | 40,56  (± 37,25) | 40,88  (± 7,05) | 16,70  (± 3.,13) | 24,03  (± 23,09) | 31,02  (± 6,76) | 0,908  (± 0,27) | 33,69  (± 16,9) |
|  | 48h | 7,84  (± 5,70) | 19,2  (± 8,96) | 5,97  (± 1,48) | 16,46  (± 8,91) | 10,79  (± 2,49) | 26,69  (± 10,14) | 16,60  (± 4,79) |
| ***E. coli***  **Bifilm E+L** | 24h | 33,95  (± 21,41) | 26,06  (± 5,89) | 26,50  (± 4,87) | 29,67  (± 26,90) | 27,19  (± 2,35) | 0,023  (± 0,019) | 9,23  (± 4,69) |
|  | 48h | 17,15  (± 0,85) | 39,50  (± 12,49) | 17,18  (± 3,48) | 43,11  (± 14,40) | 25,01  (± 5,39) | 0,00011  (± 0,00004) | 31,41  (± 14,32) |
| ***S. enterica***  **Bifilm E+S** | 24h | 63,35  (± 20,92) | 70,88  (± 33,21) | 51,33  (± 11,03) | 42,72  (± 25,26) | 71,43  (± 14,79) | 1,29  (± 0,34) | 46,92 (16,38) |
|  | 48h | 30,08  (± 11,04) | 54,52  (± 14,47) | 57,46  (± 22,51) | 64,41  (± 30,23) | 89,28  (± 18,07) | 20,00  (± 3,43) | 76,96  (± 12,91) |
| ***S. enterica***  **Bifilm L+S** | 24h | 98,48  (± 7,88) | 46,20  (± 2,17) | 45,14  (± 4,14) | 23,27  (± 15,77) | 21,18  (± 9,46) | 0,052  (± 0,039) | 50,99  (± 2,76) |
|  | 48h | 41,91  (± 20,66) | 78,33  (± 2,79) | 58,42  (± 12,54) | 67,51  (± 0,84) | 20,13  (± 2,20) | 0,00015  (± 0.00009) | 78,12  (± 21,27) |
| ***L. mon*.**  **Bifilm L+S** | 24h | 12,61  (± 2,76) | 13,12  (± 18,63) | 4,70  (± 2,00) | 2,39  (± 0,98) | 2,67  (± 0,65) | 0,015  (± 0,0079) | 16,48  (± 7,00) |
|  | 48h | 4,19  (± 2,88) | 7,00  (± 5,06) | 4,44  (± 2,84) | 4,98  (± 3,00) | 1,95  (± 1,38) | 0,00009  (± 0,00010) | 5,58  (± 3,86) |
| ***L. mon.***  **Bifilm E+L** | 24h | 19,20  (± 7,07) | 37,79  (± 10,00) | 14,66  (± 8,71) | 37,60  (± 25,68) | 27,41  (± 17,26) | 0,045  (± 0,037) | 11,30  (± 3,28) |
|  | 48h | 0,84  (± 0,66) | 17,25  (± 9,14) | 30,87  (± 3,79) | 11,01  (± 7,60) | 48,47  (± 17,19) | 0,00007  (± 0,00003) | 40,00  (± 1,67) |
